# Red Wine Extract Inhibits VEGF Secretion and Its Signaling Pathway in Retinal ARPE-19 Cells to Potentially Disrupt AMD

**DOI:** 10.3390/molecules25235564

**Published:** 2020-11-27

**Authors:** Clarisse Cornebise, Flavie Courtaut, Marie Taillandier-Coindard, Josep Valls-Fonayet, Tristan Richard, David Monchaud, Virginie Aires, Dominique Delmas

**Affiliations:** 1 Université de Bourgogne Franche-Comté, F-21000 Dijon, France; clarisse.cornebise@gmail.com (C.C.); flavie.courtaut@gmail.com (F.C.); marie.taillandiercoindard@gmail.com (M.T.-C.); david.monchaud@u-bourgogne.fr (D.M.); virginie.aires02@u-bourgogne.fr (V.A.); 2 INSERM Research Center U1231—Cancer and Adaptive Immune Response Team, Bioactive Molecules and Health Research Group, F-21000 Dijon, France; 3 Unité de Recherche Oenologie, EA 4577, USC 1366 INRA-ISVV, F-33882 Villenave d’Ornon, France; Josep.Valls-Fonayet@U-Bordeaux.Fr (J.V.-F.); tristan.richard@u-bordeaux.fr (T.R.); 4 Institut de Chimie Moléculaire (ICMUB), CNRS UMR6302, UBFC, F-21078 Dijon, France; 5 Centre Anticancéreux Georges François Leclerc, F-21000 Dijon, France

**Keywords:** polyphenols, red wine extract, AMD, retinal cells, ARPE-19, degenerative diseases, ocular diseases

## Abstract

Age-related macular degeneration (AMD) is a degenerative disease of the retina where the molecular mechanism involves the production of vascular endothelial growth factor (VEGF), a factor of poor prognosis of the progression of the disease. Previous studies have shown that resveratrol, a polyphenol of grapevines, can prevent VEGF secretion induced by stress from retinal cells. Considering the fundamental role played by VEGF in development and progression of AMD, we investigate the potential effect of red wine extract (RWE) on VEGF secretion and its signaling pathway in human retinal cells ARPE-19. To examine the effect of RWE in ARPE-19, a quantitative and qualitative analysis of the RWE was performed by HPLC MS/MS. We show for the first time that RWE decreased VEGF-A secretion from ARPE-19 cells and its protein expression in concentration-dependent manner. RWE-induced alteration in VEGF-A production is associated with a down of VEGF-receptor 2 (VEGF-R2) protein expression and its phosphorylated intracytoplasmic domain. Subsequently, the activation of kinase pathway is disturbing and RWE prevents the phosphorylation of MEK and ERK 1/2 in human retinal cells ARPE-19. Finally, this study sheds light on the interest that the use of polyphenolic cocktails could represent in a prevention strategy.

## 1. Introduction

Since the last decade, several epidemiological studies have shown an inverse relation between the incidence of coronary diseases and wine consumption, compared to wine abstinence [1,2]. In France, despite of a fat-containing diet, the incidence of coronary heat diseases is lower than other western countries with a similar diet, which is partly attributed to the moderation consumption of red wine [3]. This apparent discrepancy is called the “French paradox”. However, since the 1990s, some studies have shown that this “French paradox” is more likely resulting from a Mediterranean-type diet [4,5].

In this context, we have previously shown in a controlled environment in hospital that a moderate consumption of red wine (250 mL/day), even for a short period (2 weeks), associated with a ‘‘Western prudent’’ diet, improves various blood parameters in the lipid and antioxidative status in patients with previous coronary ischemic accidents in comparison to patients receiving water [6]. This ‘‘Western prudent’’ diet has also been proposed to prevent other pathologies such as degenerative diseases or diseases linked to oxidative stress or even cancer. Very recently, we were able to demonstrate that a red wine extract (RWE) made it possible to act on inflammation by reducing the level of certain proinflammatory cytokines produced by immune cells [7] but also by reducing the formation of an inflammatory complex in macrophages such as NLRP3 (NOD-like receptor family, pyrin domain containing 3) [8], or to reduce intestine polyp preneoplasia development in mice [9]. These effects have been confirmed in other studies, where RWE can reduce tumoral C26 growth in BALB/c mice and vascular endothelial growth factor (VEGF) [10]. The latter factor is not only important for the neoangiogenesis, which is necessary for tumor growth and the spread of metastatic cells, but it is also involved in other disease processes such as age-related macular degeneration (AMD).

Indeed, AMD is a degenerative disease of the retina characterized by progressive loss of central vision. This disease selectively affects the macula, which is the central region of the retina, which explains why only the central vision is affected, and not the peripheral vision. The formation of new blood vessels through the membrane destroys the cells lining the back of the retina. These cells serve as an anchor for the cones and rods, which allow vision, their destruction leads to a localized and permanent loss of this vision. Thus the overexpression of this vascular factor is a factor of poor prognosis of the progression of the disease and many strategies are put in place to counter the production of VEGF in particular the use of anti-VEGF antibodies. Alongside these pharmacological strategies for which resistance sometimes appears, numerous studies have been able to show the influence of nutrition on the occurrence of this type of pathology and its evolution [11,12]. Among natural compounds, polyphenols could present interest to counteract AMD. Indeed, some studies have performed in order to prove the effect of several preparations enriched with polyphenol grape extract or wine polyphenols on age-related degenerative diseases [13] and ocular diseases [14,15,16]. More specifically, we have shown that resveratrol, a polyphenol of grapevines, prevented VEGF secretion induced by oxysterols from human retinal cells [15]. However, what about a more polyphenolic complex mixture such as red wine, which contains a wide variety of polyphenols? Considering the fundamental role played by VEGF in development and progression of AMD, we investigated the potential effect of a red wine extract (RWE) on VEGF-A secretion in human retinal cells ARPE-19 having an AMD phenotype. The main goal is to determine whether RWE could decrease VEGF-A secretion and alter this signaling pathway and thus influence the progression of AMD. We show here for the first time that RWE inhibits VEGF-A secretion in a dose-dependent manner in human retinal cells. This reduction is associated with a decrease in VEGF-A protein expression. Very interestingly, RWE affects the signaling pathway leading VEGF production, particularly, RWE decreases activation of the receptor to VEGF-A, VEGF-R2 and the associated-protein kinases such as the mitogen-activated protein kinase (MEK) and extracellular regulated kinase 1/2 (ERK 1/2). 

## 2. Results

### 2.1. Qualitative and Quantitative Analysis of RWE and Its Toxicity on ARPE-19 Retinal Cells

**Figure 1:**
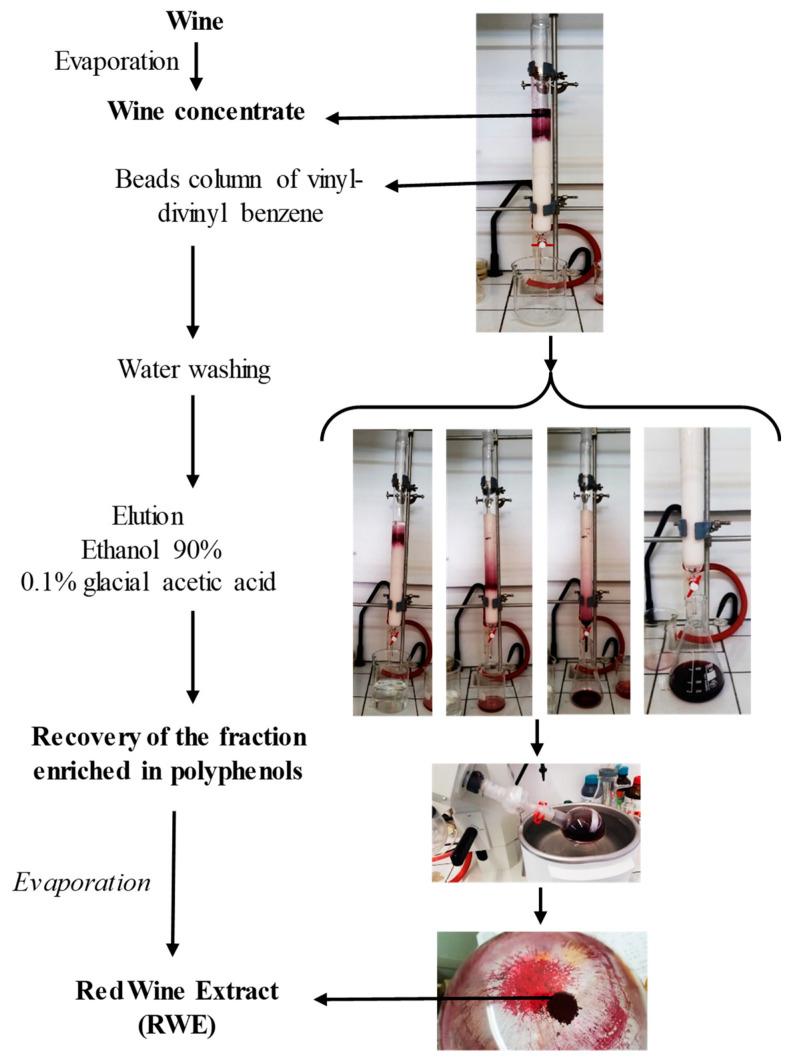
A preparative column was used to adsorb phenolic compounds present in red wine, and after alcohol evaporation, the concentrated residue was lyophilized to be finally sprayed in order to obtain the phenolic extract dry powder.

The wine vinification processes can affect, by many factors, the wine. Indeed, the climate, the vine, the country and the year can alter the quantity and the quality of polyphenols in wine, which also varies between white and red wines. Red wine present a higher quantity of polyphenols estimated to be around 900–2500 mg/L in contrary to white wine composition estimated to be around 190–290 mg/L. As we previously demonstrated in a previous study, the quantitative and qualitative wine composition of bioactive molecules such as polyphenolic compounds is essential in the biological effects that can be observed whether there are antagonistic or synergistic [9]. The Figure 1 summarizes the extraction of red wine (Santenay 1er cru Les Gravières 2012 (Côte d’Or, France), to obtain a power of red wine namely red wine extract (RWE), which was diluted in 70% ethanol at a rate of 100 mg/mL.

**Figure 2:**
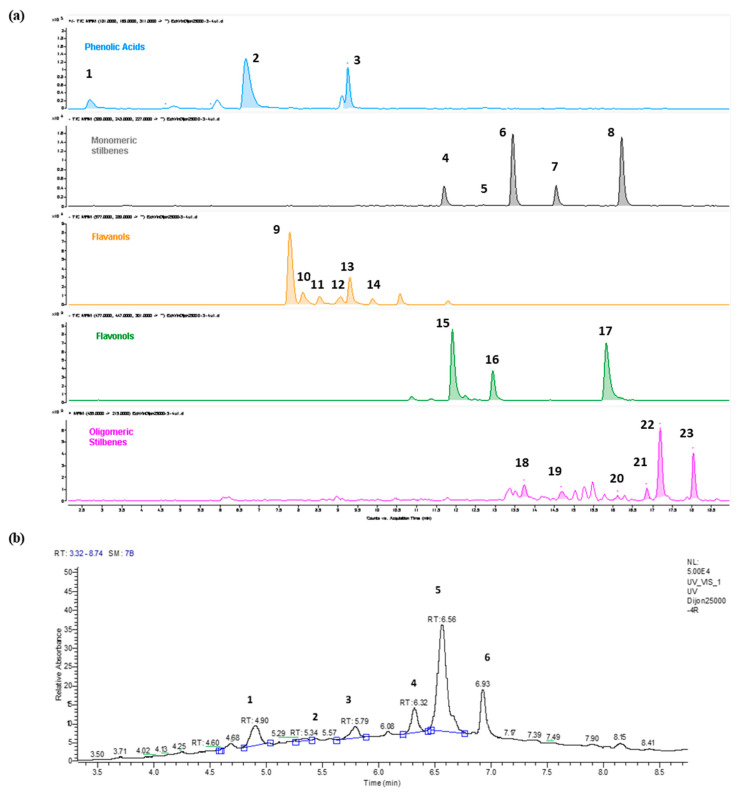
(**a**) Extracted ion chromatogram of the MRM transitions belonging to the main phenolic non-anthocyanin compounds detected in the wine extract. **1** = Gallic Acid; **2** = Caftaric Acid; **3** = Caffeic Acid; **4** = *t*-Piceid; **5** = *t*-Piceatannol; **6** = *c*-Piceid; **7** = *t*-Resveratrol; **8** = c-Resveratrol; **9** = Procyanidin B1; **10** = Procyanidin B3; **11** = Catechin; **12** = Procyanidin B4; **13** = Procyanidin B2; **14** = Epicatechin; **15** = Quercetin-3-glucuronide; **16** = Quercetin-3-rhamnoside; **17** = Quercetin; **18** = Pallidol; **19** = Parthenocisin A; **20** = Isohopeaphenol; **21** = *c*-E-viniferin; **22** = *t*-E-viniferin, **23** = *t*-w-viniferin. (**b**) UV520 Chromatogram of the main anthocyanins detected in the red wine extract. **1** = Delphinidin 3-glucoside; **2** = Cyanidin 3-glucoside; **3**= Petunidin 3-glucoside; **4**= Peonidin 3-glucoside; **5**= Malvidin 3-glucoside; **6** = Malvidin acylated derivative.

A qualitative and quantitative analysis performed by HPLC of the RWE was carried out in order to determine its polyphenolic content. As illustrated in Figure 2, we obtained different spectra allowing identifying with MRM transitions and by UV (λ = 520 nm) the main compounds contained in the extract by differentiating the main phenolic non-anthocyanin compounds (Figure 2a) and the main anthocyanins (Figure 2b).

**Figure 3:**
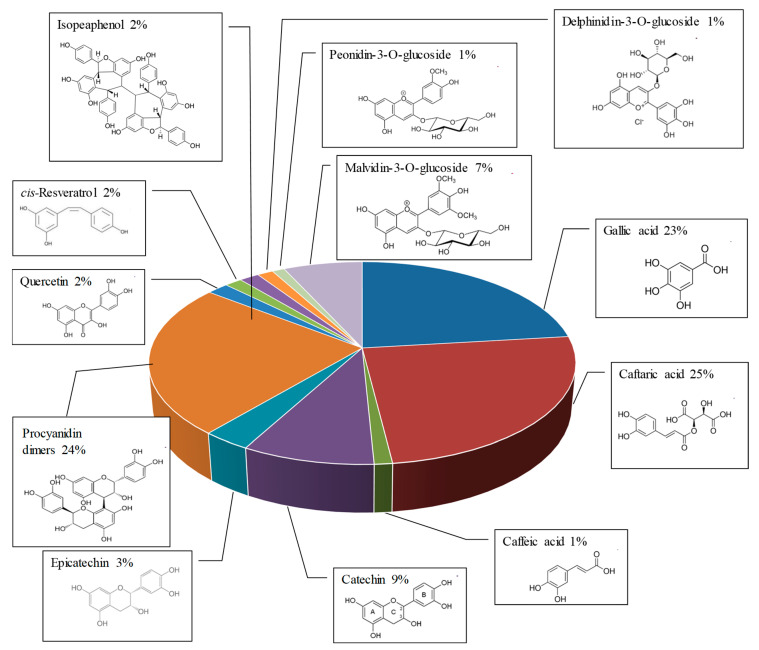
Quantitative analysis of red wine extract content and chemical structures of the main polyphenolic compounds.

The qualitative and quantitative determination of polyphenolic compounds in RWE is crucial to evaluate its biological activities since we have previously demonstrated that composition of bioactive compounds of the wine could impact its antiproliferative and anti-inflammatory activities [7,8]. In fact, we have shown that an association of resveratrol and quercetin can have a synergetic effect against the proliferation of colon cancer cells, which is not the case for other combinations such as resveratrol/catechin or resveratrol/catechin/quercetin [9]. The evaluation of the polyphenolic content revealed an important proportion of phenolic acids and flavan-3-ols. Indeed, we identified 49% of phenolic acids (gallic acid, caftaric acid and caffeic acid) and 36% of flavan 3-ols (catechin, epicatechin, procyanidins B1, B2, B3 and B4) of the total content (Figure 3). To this is added in a smaller but not insignificant quantity, 9% of anthocyanins (delphinidin 3-glucoside, cyanidin 3-glucoside, petunidin 3-glucoside, peonidin 3-glucoside and malvidin 3-glucoside); 2% of flavonols (quercetin, quercetin-3-glucoside and quercetin-3-rhamnoside) and 4% of stilbenes (*cis*- and *trans*-resveratrol, *cis*- and *trans*-piceid, *trans*-piceatannol, ε-viniferin, Ω-viniferin, pallidol and isohopeaphenol).

**Figure 4:**
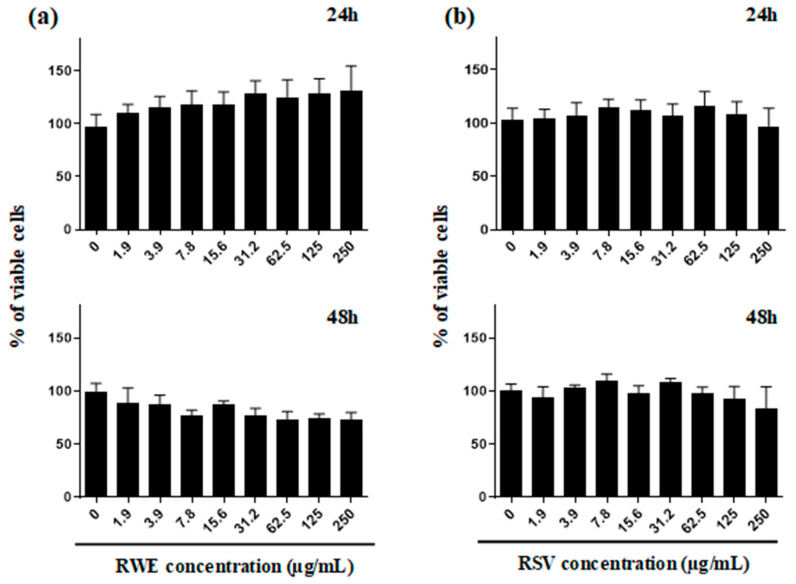
Safety assessment of red wine extract (RWE) and RSV on human retinal cell line ARPE-19. Crystal violet staining was performed in order to analyzed the cell viability of ARPE-19 after 24, 48 and 72 h of the (**a**) RWE treatment (starting concentration up to 250 µg/mL, 1:2 serial dilutions) and (**b**) RSV (starting concentration up to 250 µg/mL, 1:2 serial dilutions). Data are expressed as mean percentages ± s.d. of three independent experiments.

To specify the potential role of RWE in AMD and more particularly against VEGF, we first evaluated its toxicity on undifferentiated ARPE-19 retinal cells mimicking cells affected by AMD [17]. As revealed by cytotoxic curves that whatever the time of treatment, 24 and 48 h, RWE had no significant impact on the cellular viability of human retinal cells ARPE-19 with a game of increasing concentrations of RWE to 0 up to 250 µg/mL (Figure 4a). In the same manner, resveratrol (RSV) used in our experiment as a reference compound, presented no significant toxicity at the time of treatment and concentrations used (Figure 4b). In the following experiments, RWE was therefore used at three concentrations namely 30, 50 and 100 µg/mL in ARPE-19 cell lines. These concentrations were chosen both because they are noncytotoxic on retinal cells and because we have previously shown with these same concentrations that RWE was able to present significative properties (i.e., inhibition of proinflammatory cytokines production from macrophages [8], prevention of naïve T lymphocytes differentiation into proinflammatory T helper 17 cells [7] and inhibition of polyps development [9]). Furthermore, the secretion of VEGF by retinal cells being a rapid process with an early response of the kinase cascade activation, we explored the effect of RWE after 24 h of treatment of APRE-19 cells. In this experiment, RSV was used as a comparison at a concentration of 30 µM, which is non cytotoxic on the cell line ARPE-19. Indeed, we have previously shown a protective effect of RSV against toxic effects of oxysterols in retinal cells after 24 h and 48 h of treatment [15].

### 2.2. RWE Prevents VEGF Secretion from ARPE-19 Cells and Its Protein Expression

**Figure 5:**
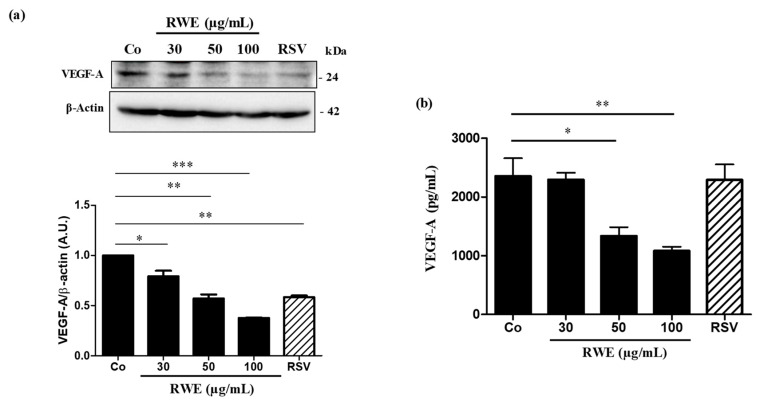
RWE decreases VEGF-A protein expression and its secretion from ARPE-19 cells. (**a**) Upper panel: representative immunoblot of VEGF6A protein expression from three independent experiments in human retinal ARPE-19 cells after 24 h of treatment without (Co) or with 30, 50 and 100 µg/mL of RWE or with RSV 20 µM. β-actin was used as a loading control. Down panel: densitometry quantification of Western blotting. Data are expressed as the mean folds’ induction ± SEM of three independent experiments. *p* values were determined by a one-way ANOVA followed by Tukey’s multiple comparison test. * *p* < 0.05, ** *p* < 0.01 and *** *p* < 0.001. (**b**) As in (a) VEGF-A secretion was measured in the cell medium by ELISA. The data are the mean ± S.D. of four independent experiments with *n* = 10. *p* values were determined by a one-way ANOVA followed by Tukey’s multiple comparison test. * = *p* < 0.05; ** = *p* < 0.01; *** = *p* < 0.001.

Since VEGF is the main factor involved in the neoangiogenesis and the progression of AMD, we firstly determined whether RWE was able to affect its expression. Immunoblotting analysis shown that after 24 h of treatment, RWE strongly decreased VEGF protein expression in ARPE-19 cells in a concentration-dependent manner as compared to the control (Figure 5a). Interestingly, RWE at 50 µg/mL presented the same inhibitory effect of VEGF expression as RSV at 30 µM, and a more effect at 100 µg/mL (Figure 5a). These results suggested that polyphenolic compounds present into RWE at lower concentrations act in a synergistic manner to decrease VEGF expression. This is in agreement with what we previously found regarding the effect of RWE in other models such as macrophages or immune cells [7,8]. This decrease in VEGF protein expression by RWE should normally be accompanied by a decrease in VEGF secretion by retinal cells. In order to verify this hypothesis, we quantified using an ELISA method, the levels of VEGF released into the culture medium. As assumed, 24 h of treatment, RWE decreased the levels of VEGF in a very significant manner for the concentrations of 50 and 100 µg/mL (Figure 5b). Surprisingly, the RSV at the concentrations of 30 µM after 24 h of treatment failed to decrease the production of VEGF in retinal cells.

### 2.3. RWE Prevents VEGF-A Secretion from ARPE-19 Cells and Its Protein Expression

**Figure 6:**
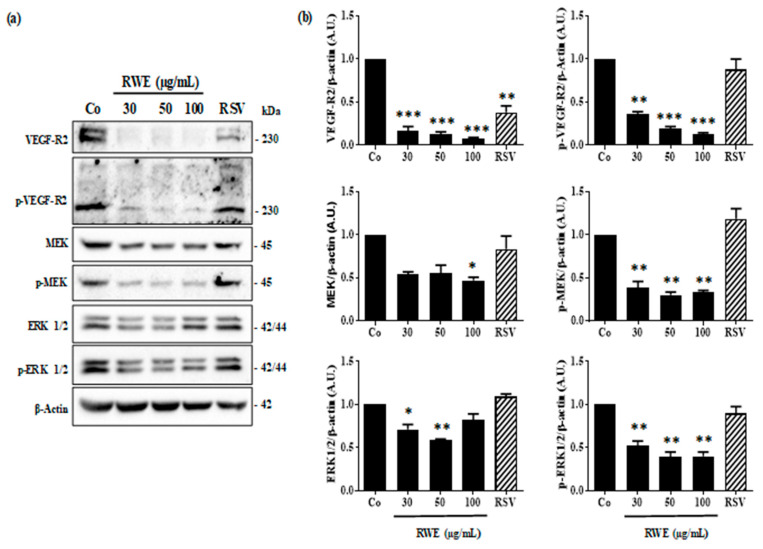
RWE disrupts VEGF-R2 kinase activation pathways. (**a**) Immunoblotting analysis of VEGF-R2, phospho VEGF-R2 (*p*-VEGF-R2), MEK, phospho MEK (*p*-MEK), ERK 1/2 and phospho-ERK 1/2 (*p* ERK 1/2) in RWE-treated ARPE-19 cells with increasing concentration (30, 50 and 100 µg/mL) or with RSV (20 µM) for 24 h. β-actin was used as a loading control. (**b**) Densitometry quantification of Western blotting. Data are expressed as the mean folds induction ± SEM of three independent experiments. *p* values were determined by a one-way ANOVA followed by Tukey’s multiple comparison test. * *p* < 0.05, ** *p* < 0.01 and *** *p* < 0.001.

Secretion of VEGF-A by retinal cells results from the activation of the signaling pathway involving VEGF-specific tyrosine kinase receptors whose activation loop results from a phosphorylation cascade through the induction of successive kinases [18]. Indeed, the binding of VEGF-A occurs mainly on the VEGF-R2 receptor, which induces its phosphorylation and the intracytoplasmic signaling cascade, passing through the phosphorylation of the mitogen-activated protein kinase kinase (MEK) protein, and with the ultimate activation of the phosphorylation of the extracellular regulated kinase 1/2 (ERK 1/2) protein. The latter once phosphorylated could then activate various nuclear transcription factors making it possible to activate the gene coding for VEGF. We observed by immunoblotting that RWE since low concentration at 30 µg/mL was able to strongly inhibit VEGF-R2 protein expression and its phosphorylated form (Figure 6). This disruption in VGEF-R2 activation through its intracytoplasmic phosphorylation leads to a decrease of activation of the MEK and ER ½ pathway. As shown by the immunoblotting 24 h of treatment with RWE decreased significantly the phosphorylation of MEK and ERK 1/2 in ARPE-19 cells (Figure 6). Although RSV decreased significantly the expression of VEGF-R2, the polyphenol failed to prevent the phosphorylation of VEGF-R2 and subsequently then allowed the phosphorylation cascade to take place.

## 3. Discussion

**Figure 7:**
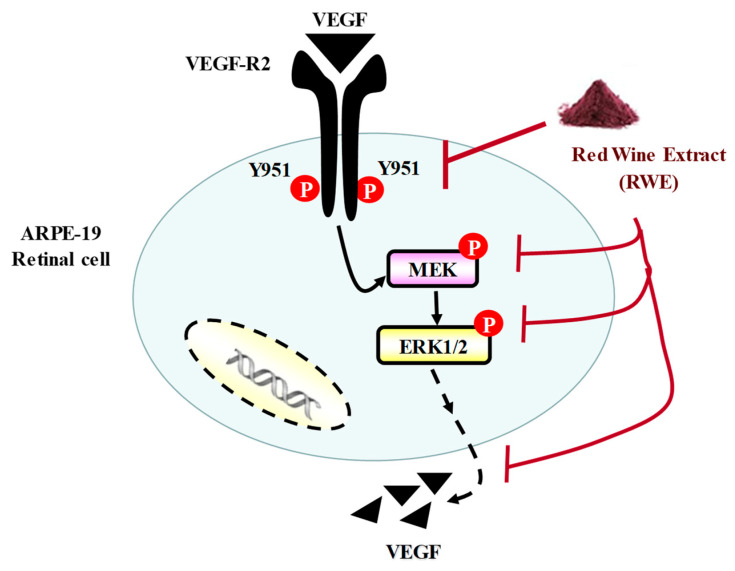
RWE prevents VEGF production by disruption of VEGF-R2 activation. RWE decreases the phosphorylation of VEGF-R2 (P-VEGF-R2) and subsequently prevents the phosphorylation of MEK (P-MEK) and ERK 1/2 (P-ERK 1/2) in human retinal cells ARPE-19.

AMD is a multifactorial degenerative pathology, which results from the conjunction of several risk factors. The most important of these is age, with a sharp increase after the sixth decade, but there are also genetic, ethnic or environmental factors. Indeed, smoking, obesity and diet are linked to this disease [19]. Studies have shown that in developed countries, AMD is the leading cause of visual impairment and blindness affecting people over 65 years [20]. The molecular mechanism of AMD involved a growth factor, namely VEGF-A, for which the secretion induces the appearance of new immature and poor quality blood vessels that invade the different histological layers that make up the retina. This results in the destruction of the thin membrane separating the retina from the bloodstream [21]. This invasion combined with the fact that these vessels, of poor quality, allow serum to diffuse causes a progressive loss of vision by the destruction of the underlying cells. It therefore appears essential to control the secretion of VEGF in order to limit the phenomenon of neoangiogenesis and thus limits the disappearance of retinal cells. To overcome this VEGF-A secretion by retinal cells affected by AMD, there is a growing research interest to develop various antibodies against VEGF-A [22], but this strategy failed in one form of AMD, the dry form for which there is no treatment at present apart from recommendations in particular in terms of nutrition or supplementation [23]. Moreover, some resistance to anti-VEGF-A antibodies is highlighted in patients with an exudative form [24]. Thus some studies have tried to demonstrate the action of nutrition or supplementation against VEGF-A secretion from retinal cells such as oral nutritional supplementation in patients with intermediate or advanced AMD, where the risk of vision loss of three or more line was reduced by 19% with this supplementation [11]. In this way, we tested the ability of a polyphenol-enriched extract (RWE) from a red wine to act on the VEGF-A pathway in human retinal cells, which is an AMD phenotype. The present study highlights that RWE was able to prevent secretion of VEGF-A from ARPE-19 cells in a dose-dependent manner, which is associated with a decrease of VEGF-A protein expression. Usually, its activity is linked to its binding to the surface of tyrosine kinase receptors (VEGF-R or vascular endothelial growth factor receptor). The binding is done on the extracellular part of the transmembrane receptors. There are three isoforms of receptors that bind VEGFs with different affinities: VEGF-R1, VEGF-R2 and VEGF-R3. Each of them activates different signaling channels resulting in different effects. It has thus been possible to demonstrate the role of VEGF-R1 and VEGF-R2 in the process of angiogenesis, while that of VEGF-R3 is more linked to lymphangiogenesis [25]. More generally, VEGF-A binds to VEGF-R1 and VEGF-R2. These have 44% homology in their sequence, but their affinity for their ligand differs, and they induce different cellular and biological effects. The receptor with the strongest affinity for VEGF-A is VEGF-R1, but the receptor predominantly present on the surface of epithelial cells is VEGF-R2, so it is the latter that appears to be the main mediator of angiogenic activity. In addition, VEGF-R1 has been shown to have lower activity due to the presence of an inhibitory sequence, the absence of which in VEGF-R2 leads to an improvement in tyrosine kinase activity. VEGF binding to its receptor leads to phosphorylation of its intracytoplasmic domain for engaging the mitogen-activated protein kinase (MAP kinase) activation cascade including MEK and ERK (Figure 7).

We show in the present report that RWE was able to alter the phosphorylation of intracytoplasmic domain of VEGF-R2 leading a disrupting in kinase pathway (Figure 7). By disrupting the phosphorylation of MEK and ERK in retinal cells, RWE could alter the pathway inducing the VEGF production.

It is very surprising that the polyphenol RSV alone was not able to prevent this pathway at 20 µM. This absence of effect on VEGF secretion by retinal cells was with a lack of effect on the MAPK activation pathway. This could be explained by the fact that RWE contains many polyphenolic compounds that, even at very low concentrations, could act in synergy in order to exert an action against the activation of the VEGF-R2 pathway. Analysis of polyphenolic composition revealed a high content of phenolic acid (49%) and flavan 3-ols (36%). These compounds were particularly important since separately they were shown to exert an action on VEGF-A production. For example, among phenolic acids, gallic acid exerted an antiangiogenic effect in ovarian cancer cells [26] or in vascular smooth muscle cells [27], and caffeic acid reduces the VEGF secretion in human retinal pigment epithelial cells under hypoxic conditions [28]. Similarly, delphinidin has been shown to inhibit angiogenesis through the suppression of VEGF expression in lung cancer cells [29]. The presence of quercetin could explain a potential mechanism of synergism. Indeed, resveratrol + quercetin association increases resveratrol uptake by colon carcinoma cells, and in combination with RWE this combination increases the influx of resveratrol [9,30,31]. In a similar manner, the presence of quercetin in RWE could increase the uptake of some polyphenols and favors the synergism between the bioactive compounds to potentiate their action against neoangiogenesis in AMD.

## 4. Materials and Methods

### 4.1. Cell Lines and Cell Culture

The human retinal pigmented epithelial cell line ARPE-19, purchased from the American Type Culture Collection (Manassas, VA, USA), were maintained in Dulbecco’s modified Eagle’s F12 medium (DMEM/F12) supplemented with 10% fetal bovine serum (Dutscher, Brumath, France), 1% penicillin/streptomycin in a humidified atmosphere of 5% CO_2_ at 37 °C. Cells were seeded and grown to a subconfluence of 60–70% in normoxia. Twenty four hours after seeding, the medium was removed and the cells were washed once with Hank’s Balanced Salt Solution (HBSS, Dutscher, Brumath, France) before reincubating in DMEMF12 with 1% FBS and 1% penicillin/streptomycin. The following day, cells were treated with DMSO, RWE with indicated concentrations or resveratrol 20 µM.

### 4.2. Chemical Reagents and Antibodies

Resveratrol (RSV) was purchased from Sigma-Aldrich (St. Quentin Fallavier, France) and dissolved in ethanol 70%. VEGFR2 (sc-2479, 1:1000); *p*-VEGFR2 TYR951 (sc-4991, 1:1000); ERK1/2 (sc-4695, 1:1000); *p*-ERK1/2 (sc-9101, 1:1000); MEK1/2 (sc-91226, 1:1000) and *p*-MEK1/2 (Ser221; sc-2338, 1:1000) were obtained from Santa Cruz Biotechnology (Nanterre, France) and *p*-VEGFR2 (TYR 1054; 267,398, 1:1000) was obtained from EMD Millipore Corporation. Β-Actin (A1978, 1:5000) was obtained from Abcam. (Paris, France). (+)-Catechin (>99%), (−)-epicatechin (>99%), procyanidin B1 (>90%), procyanidin B2 (>90%) and quercetin as a dihydrate (>99%) were obtained from Extrasynthese (Genay, France). Gallic acid (>97.5%) and caffeic acid (>98%) were obtained from Sigma-Aldrich (St Quentin Favrallier, France). Caftaric acid (>98%) was purchased from Carl Roth (Karslruhe, Germany). E-resveratrol, hopeaphenol and malvidin 3-glucoside were purified as standards in the MIB laboratory using a Varian Pro Star preparative HPLC. Purity was assessed to be over 90% by HPLC.

### 4.3. Preparation of the Red Wine Extract

The red wine extract (RWE) was obtained from French red wine, Santenay 1er cru Les Gravières 2012 (EARL Capuano-Ferreri Santenay, Côte-d’Or, France) selected by BIVB (Bureau Interprofessionnel des Vins de Bourgogne, Beaune, France) and provided by CTIVV (Centre Technique Interprofessionnel de la Vigne et du Vin, Beaune, France). Red wine extract dry powder was prepared and analyzed as previously described [7,8]. Briefly, the phenolic compounds contained in the wine were separated from the liquid using an absorption column and solubilized in alcohol. After evaporation of the alcoholic eluent using a rotary evaporator, the concentrated residue was deposited on the column (Diainon^®^ HP-20, Supelco, Germany). For the retention phase, the column reservoir was filled with distilled water, and the flow rate was adjusted to approximately 20 drops/min. Then the polyphenol fraction retained was eluted using a solution of ethanol and 0.1% glacial citric acid, and the flow rate was adjusted to about 40 drops/min. The fractions collected after elution were concentrated to dryness with a rotary evaporator. In this way, we obtained, from 1 L of red wine, 104 g of phenolic extract in powder form, containing 5.04 mg g^−1^ of total phenolic compounds expressed as the gallic acid equivalent.

### 4.4. High-Performance Liquid Chromatography Analysis

Triplicates of 5 mg freeze-dried extract was dissolved with 200 µL of a mixture water:methanol (1:1) and centrifuged for 5 min at 10,000× *g* before anthocyanins and polyphenols analysis. Anthocyanins were analyzed with a Thermo Scientific Vanquish UHPLC equipped with a Thermo Scientific MWL detector operating at 520 nm. Of the sample 1 µL was injected in Agilent Zorbax SB-C18 (100 mm × 2.1 mm × 1.8 µm) column at 35° using the following conditions of separation: solvent A (5% formic acid in MilliQ water) and solvent B (5% formic acid in acetonitrile); flow: 0.35 mL/mi and gradient: 2.5% B (0–1 min), 17% B (5–7 min), 45% B (10–11 min), 95% B (11–12.5 min) and 2.5% B (13–15 min). A calibration curve in the range of 6.25–80 mg/L was built with malvidin 3-glucoside previously purified in our laboratory. The 5 quantified anthocyanins (delphinidin 3-glucoside, cyanidin 3-glucoside, petunidin 3-glucoside, peonidin 3-glucoside and malvidin 3-glucoside) were quantified as malvidin 3-glucoside. The rest of the polyphenols was analyzed by HPLC–MS/MS methodology previously published [32] with slight modifications. The compounds were separated with an Agilent 1260 HPLC instrument. Of samples 4 µL were eluted on an Agilent Zorbax SB-C18 (100 mm × 2.1 mm × 1.8 µm) column at 40 °C with a binary solvent system of solvent A (0.1% formic acid in water) and solvent B (0.1% formic acid in acetonitrile). The chromatographic separation was conducted with a flow rate of 0.4 mL/min and the following gradient: 10–18% B (0–1 min), 18–33% B (1–6.5 min), 33% B (6.5–9.5 min), 33–40% B (9.5–15 min), 40–90% B (15–16 min), 90% B (16–19 min) and 90–10% B (19–20 min). The HPLC was coupled to an Agilent 6430 Triple Quadrupole mass spectrometer, which operated under the following parameters: alternate positive/negative mode; drying gas (nitrogen), 11 L/min; nebulizer pressure, 15 psi; temperature, 350 °C and capillary voltage, 3000 V. Specific MRM transitions were used for the detection and quantification of each compound. Calibration curves were established with pure standards in the range of 0.03–15.00 mg/L except for catechin, gallic acid and caftaric acid (range 0.03–100 mg/L). All compounds were quantified as their corresponding standard except flavan 3-ol dimers B3 and B4, which were expressed as dimer B1 and B2 respectively, and isomers *c*-resveratrol and c-piceid, which were determined as their respective *t*-isomer.

### 4.5. Cell Viability Assays

The viability assays were assessed by crystal violet staining (Sigma Aldrich, St. Quentin Fallavier, France). Retinal ARPE-19 cells were seeded into 96-well plates after 24 h, the medium was replaced by new medium containing different concentrations of RWE or RSV and incubated for 24, 48 and 72 h. Then, cells were washed with phosphate-buffered saline (PBS) and fixed with ethanol for 10 min at 4 °C. Finally, cells were stained with a crystal violet solution (0.5% (*w*/*v*) crystal violet in 25% (*v*/*v*) methanol) for 15 min at room temperature, then the absorbance was measured at 590 nm using a Biochrom Assays UVM 340 microplate reader, following extraction of the dye using an acetic acid 33% solution.

### 4.6. Measurement of VEGF Secretion

Cell culture media were saved from the final 24 h of treatment of ARP-19 with RWE (30, 50 or 100 µg·mL^−1^) or RSV (20 µM). VEFG levels in cell culture conditioned medium were measured using the enzyme-linked immunosorbant assay (ELISA; BMS277-2 eBioscience) with antibodies mainly specific to VEGF-121; VEGF-165 and VEGF-189.

### 4.7. Immunoblotting Analysis

ARPE-19 cells were treated as described above. Next, cells were collected and lysed in radioimmunoprecipitation assay (RIPA) buffer (50 mM Tris-HCl, 150 mM sodium chloride, 0.1% sodium dodecyl sulfate, 0.5% sodium deoxycholate, 1% NP40 and pH 8) containing a protease inhibitor, phenylmethylsulfonyl fluoride (PMSF; 100 μM, Sigma-Aldrich, St. Quentin Fallavier, France), phosphatase inhibitor, sodium fluoride (50 mM) and a protease inhibitor cocktail (Roche, Boulogne-Billancourt, France). Protein concentrations were measured using the QuantiPro™ BCA (Bicinchoninic Acid; Sigma Aldrich, St. Louis, MO, USA; bovine serum albumin (BSA) was used as a standard). Fifty micrograms of proteins were prepared in the Laemmli gel loading buffer (50 mM Tris-HCl, 10% glycerol, 5% 2-mercaptoethanol, 2% sodium dodecyl sulfate, pH 6.8 and 0.1% bromophenol blue). After being boiled 5 min at 95 °C, samples were loaded and separated on sodium dodecyl sulfate–polyacrylamide gel electrophoresis (SDS-PAGE). Protein size markers (Thermo Fisher Scientific, Illkirch-Graffenstaden, France) were loaded without heating. Then, proteins were separated on sodium dodecyl sulfate–polyacrylamide gel electrophoresis (SDS-PAGE). Proteins separated on gels were transferred to nitrocellulose membrane (Amersham, Les Ulis, France). Membranes were blocked for 1 h at room temperature in either 5% of bovine serum albumin (BSA) or 5% of skimmed milk powder dissolved in PBS-T (PBS containing 0.1% Tween 20) and incubated with the primary antibody on a rocker platform at 4 °C overnight. Primary antibodies for Western blot listed in chemical reagents and antibodies were diluted with 5% *w/v* non-fat milk or 5% BS PBS-T. After three 10 min in PBS-T, primary antibodies were detected using appropriate horseradish peroxidase (HRP)-conjugated secondary antibodies (Jackson ImmunoResearch, Interchim, Montlucon, France) for 1 h at room temperature, followed by exposure to enhanced chemiluminescence (ECL; Bio-Rad, Marnes-la-Coquette, France). Detection of immunoreactive bands was performed by ChemiDoc^TM^ XRS + imaging system (Bio-Rad, Marnes-la-Coquette, France), and blots were analyzed with Image Lab^TM^ version 6.0.1 software (Bio-Rad).

### 4.8. Statistical Analysis

Statistical analysis was conducted using the GraphPad6.0 Prism software (GraphPad Software, La Jolla, San Diego, CA, USA). Data are represented as means ± standard deviation (SD) for triplicate assay samples (otherwise mentioned), of at least three independent experiments. The difference between mean values was determined by the multiple Student’s *t* test or by Mann–Whitney *U* test. All *p* values are two-tailed; *p* < 0.05 was considered significant (* *p*  <  0.05, ** *p*  <  0.01 and *** *p*  <  0.001).

## 5. Conclusions

Increasing life expectancy will continue to increase the prevalence of age-related eye diseases in economically developed countries. Its chronic course is currently impossible to cure, but it can be delayed. In this, the search for new bioactive molecules of low toxicity could represent a major interest for the prevention or for delaying the progression of AMD. In this study we showed for the first time that a polyphenol-enriched extract, RWE, could decrease VEGF-A secretion for human retinal ARPE-19 cells mimicking the AMD phenotype. This disturbing of VEGF-A production is associated with a decrease of its protein expression. Very interestingly, RWE affects the MAP kinase pathway through downregulation of phosphorylated forms of MAK and ERK1/2 proteins. Thus the use of polyphenolic cocktails could represent a potential interest in a therapeutic strategy. Nonetheless, further studies should better clarify the role of each of the polyphenols present, but also to specify more precisely the molecular mechanisms involved and their effects in preclinical models of AMD.
